# Defect-Selective Luminescence in Hydroxyapatite Under Electron and Gallium Ion Beams

**DOI:** 10.3390/ma19020321

**Published:** 2026-01-13

**Authors:** Verónica J. Huerta, Fabián Martínez, Hanna M. Ochoa, Olivia A. Graeve, Manuel Herrera-Zaldívar

**Affiliations:** 1 Centro de Nanociencias y Nanotecnología, Universidad Nacional Autónoma de México, Ensenada C.P. 22800, Baja California, Mexico; veronicajhg@gmail.com (V.J.H.); g13_ochoa@ens.cnyn.unam.mx (H.M.O.); 2 Program in Materials Science and Engineering, University of California San Diego, La Jolla, CA 92093-0418, USA; fmm003@ucsd.edu; 3 Department of Mechanical and Aerospace Engineering, University of California San Diego, La Jolla, CA 92093-0418, USA

**Keywords:** hydroxyapatite, defect engineering, oxygen vacancies, hydroxyl vacancies, ion irradiation, electron irradiation

## Abstract

We report a defect-selective luminescence response in calcium-deficient hydroxyapatite (HAp) induced by electron and ion irradiation. Compacted HAp pellets prepared from hydrothermally grown nanofibers were investigated to analyze defect-related luminescence using photoluminescence (PL) and cathodoluminescence (CL) techniques, both before and after compaction. Low-energy electron beam irradiation (15 keV) produced a two-stage luminescent response, an initial enhancement arising from field-assisted activation of OH-channel vacancies (V_OH_ and V_OH_ + H_i_), followed by an exponential decay attributed to defect annealing. Monochromatic transient CL measurements show that this rise–decay behavior is selective to the OH-related bands at 2.57 and 2.95 eV, whereas the 3.32 and 3.67 eV emissions exhibit only a monotonic exponential decay. The corresponding decay constants further indicate that the activated OH-channel vacancies anneal more rapidly than the other centers, consistent with their higher electron-capture probability and lower structural stability. In contrast, ${\mathrm{Ga}}^{+}$ ion irradiation (30 keV, 1.4 × 10^−13^ A/µm^2^) induced progressive monotonic luminescence quenching, primarily driven by selective annealing of oxygen vacancies in $PO_{4}^{3-}$ groups. These complementary pathways, electron-induced activation and ion-driven suppression, demonstrate that irradiation serves as a versatile tool for defect engineering in hydroxyapatite. Beyond providing fundamental insights into vacancy stability, these results open new routes for tailoring the optical, sensing, and bioimaging functionalities of HAp through controlled irradiation.

## 1. Introduction

Hydroxyapatite [HAp, Ca_5_(PO_4_)_3_OH] is one of the most widely studied biomaterials due to its chemical similarity to the mineral phase of bones and teeth [1,2], excellent biocompatibility [3,4], and high bioactivity [5,6]. Beyond its established role in hard tissue engineering, HAp nanostructures have attracted increasing interest for applications in photonics [7], bioimaging [8], and drug delivery [9], where their optical properties provide added functionality [10]. A central challenge in these advanced applications is the control of intrinsic point defects, particularly oxygen and hydroxyl vacancies, which strongly influence HAp luminescence [11], charge transport [12], and even magnetic behavior [13,14].

Defect engineering has emerged as a powerful strategy to tailor the optical and functional properties of a variety of materials [15,16,17,18,19]. Recent advances have shown that point defects, particularly anion vacancies, strongly modulate the physicochemical and biological response of biomedical ceramics and nanomaterials. Vacancy-driven modifications alter their band structure, surface reactivity, catalytic activity, and optical response, enabling precise regulation of biomaterial performance. This strategy has been successfully applied to several oxide-based biomaterials, where oxygen-related vacancies enhance redox activity, luminescence sensitivity, ion release, or catalytic behavior relevant to tissue engineering and nanotheranostics [20]. In hydroxyapatite specifically, defect engineering has also been shown to regulate degradation and biological response. Krypton-ion irradiation induces structural disorder and oxygen-related vacancies that enhance dissolution kinetics and modify cell interactions without altering stoichiometry, highlighting the critical role of vacancy density in functional performance [21].

Luminescence associated with specific vacancies (V_O_ in $PO_{4}^{3-}$ groups, V_OH_ in ${\mathrm{OH}}^{-}$ channels, and their complexes) provides a sensitive probe of the defect structure, while also offering opportunities for optoelectronic and sensing applications [11]. However, approaches to selectively and controllably modify defect populations remain limited. High-energy irradiation (e.g., MeV-range electrons) has been shown to generate oxygen vacancies in bulk HAp, but it lacks fine control over defect selectivity [22]. In this context, low-energy electron beam irradiation (LEEBI) represents an attractive alternative. By delivering relatively gentle and localized energy (15 keV in this study), LEEBI enables a gradual and defect-specific activation pathway, particularly within the less stable ${\mathrm{OH}}^{-}$-sublattice. While no previous reports exist on defect-selective irradiation in hydroxyapatite, related studies in wide-band oxides have shown that electron irradiation can substantially reshape the population of luminescent centers. Investigations on Al_2_O_3_ and Al_2_O_3_:Cr have shown that high-energy electron exposure modifies F^−^ and F^+^ center concentrations, enhancing or suppressing their respective luminescence through carrier capture and energy-transfer processes between the host and impurity states [23]. This suggests that HAp may also exhibit irradiation-driven pathways amenable to controlled defect engineering.

In this study, we demonstrate that LEEBI selectively activates radiative hydroxyl-related vacancies (V_OH_), producing time-dependent luminescence enhancements that can be quantitatively modeled through competing activation and annealing kinetics. Controlled modulation of V_O_ and V_OH_ centers could thus enable opto-bio interfaces where emission intensity and color are engineered at the nanoscale. Complementarily, ${\mathrm{Ga}}^{+}$ ion irradiation produces the opposite effect, progressively quenching luminescence by annealing unstable defect populations. By contrasting activation under LEEBI with suppression under ${\mathrm{Ga}}^{+}$ ions, this dual approach highlights irradiation as a versatile tool for defect engineering in HAp, revealing two distinct pathways for defect evolution. To our knowledge, this is the first demonstration of a defect-selective luminescence response in hydroxyapatite, establishing new means to tailor its optical and functional properties for biomedical and photonic applications. By combining photoluminescence and cathodoluminescence spectroscopies with kinetic modeling, we uncover distinct irradiation pathways that open perspectives for spatially controlled defect engineering in HAp.

## 2. Materials and Methods

Hydroxyapatite [HAp, Ca_5_(PO_4_)_3_OH] was synthesized via a hydrothermal method. Two 0.1 M aqueous solutions were prepared. The first consisted of calcium nitrate tetrahydrate [Ca(NO_3_)_2_·4H_2_O, 99%, Alfa Aesar (Heysham, UK), A16645], obtained by dissolving 2.9518 g of the reagent in 125 mL of deionized water. The second was ammonium hydrogen phosphate [(NH_4_)_2_HPO_4_, 98%, Alfa Aesar, A17416], prepared by dissolving 0.9878 g of the salt in 74.8 mL of deionized water. The reagent quantities and volumes were adjusted to maintain a Ca/P ratio of 1.67. The first solution was placed in a three-neck round-bottom flask and heated to 373 K under continuous magnetic stirring, which was maintained until the end of the reaction. Once the temperature was reached, the second solution was added dropwise. The resulting mixture was stirred for 36 h at 373 K. After the reaction, the resulting white precipitate was collected by filtration and washed with deionized water until a pH of 7 was achieved. The product was dried at 323 K for 2 h and lightly ground to obtain a fine white powder. The atomic composition of the synthesized HAp powder is presented in Table 1. Detailed XPS analysis of Ca 2p, P 2p, and O 1s core levels for hydroxyapatite powders synthesized by the same method was previously reported in Ref. [11], where the defect-related chemical states were established.

To evaluate the optical properties of HAp powder after elimination of oxygen vacancies (V_O_), annealing was performed in flowing high-purity oxygen (O_2_, INFRA Co. (Mexico City, Mexico), 99.99%) at 773 K for 5 h (flow rate: 50 sccm). After annealing, the samples were maintained under O_2_ flow during cooling to prevent atmospheric contamination.

For pellet preparation, 100 mg of HAp powder was finely ground in an agate mortar. The powder was then loaded into a Specac^®^ die set (13 mm in diameter), ensuring maximum compaction to prevent fractures during pressing. The die was mounted in a manual hydraulic press (Specac, Kent, UK), and loads of 3, 4, and 5 ton-force (corresponding to approximately 2.2 × 10^8^, 3.0 × 10^8^, and 3.7 × 10^8^ N/m^2^, respectively) were applied to fabricate the HAp-P3, HAp-P4, and HAp-P5 pellets and were maintained for approximately 1 min after reaching these loads to promote uniform compaction and minimize porosity (the 3, 4 and 5 in the name correspond to the loads applied in ton-force). The compacted pellets were subsequently sectioned into four equal parts using a pellet cutter, and each piece was mounted on a Si (100) substrate with silver paint to provide electrical contact.

The crystalline structure of the as-synthesized HAp powder and compacted pellets was analyzed by X-ray diffraction (XRD, Philips X’Pert MPD (Almelo, The Netherlands)) operated at 30 kV and 10 mA using CuKα radiation (Kα_1_ + Kα_2_, λ ≈ 0.15405 nm) with K_β_ suppression provided by a Ni filter. Diffraction patterns were collected over a 2θ range from 20 to 70° with a step size of 0.02°. Vibrational modes were examined by Raman spectroscopy (Horiba Jobin-Yvon (Longjumeau, France), LabRam HR800 micro-Raman system) using a 633 nm He-Ne laser. The same instrument was also used to obtain photoluminescence (PL) spectra in the 300–1100 nm range, excited with a 325 nm He-Cd laser. The elemental composition and Ca/P atomic ratio were determined by energy dispersive spectroscopy (EDS, Oxford Instruments X-Max, 20 mm^2^ detector, Oxford, UK) attached to a JEOL JIB-4500 SEM (Tokyo, Japan). The morphology of the HAp nanostructures was examined using a transmission electron microscope (TEM, JEOL JEM-2200FS + CS, Tokyo, Japan) operated at 200 kV. Surface composition was analyzed by X-ray photoelectron spectroscopy (XPS, SPECS system, Berlin, Germany) equipped with a PHOIBOS WAL analyzer (Berlin, Germany) and an Al anode. Survey spectra were acquired with a 150 eV pass energy and 1 eV step size. Spectra were calibrated using the C 1s peak at 284.6 eV and processed with CasaXPS software (version 2.3.24PR1.0). Cathodoluminescence (CL) spectra in the 200 to 800 nm range were collected using a Gatan MonoCL4 system integrated into the JEOL JIB-4500 SEM. HAp pellets were irradiated with an electron beam at current densities of 1.0 × 10^−11^ and 2.2 × 10^−11^ A/mm^2^. In addition, ${\mathrm{Ga}}^{+}$ ion irradiation was performed using a focused ion beam (FIB) system at 30 keV, with a current density of 1.4 × 10^−13^ A/mm^2^ and exposure times of 5, 10, 15, and 20 min.

## 3. Results and Discussion

### 3.1. Crystallinity and Morphology

**Figure 1:**
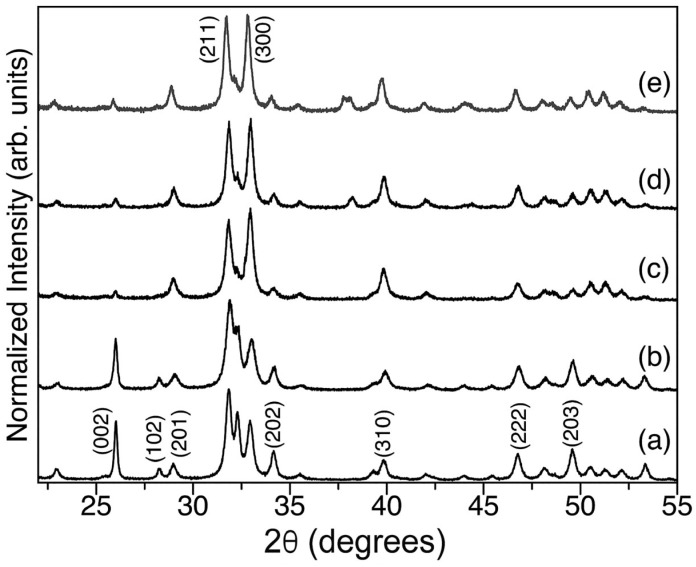
XRD patterns of the (**a**) HAp powders and (**b**) HAp powder annealed at 773 K in O_2_ atmosphere, (**c**) HAp-P3, (**d**) HAp-P4, and (**e**) HAp-5 pellets, confirming the hexagonal hydroxyapatite structure.

**Figure 2:**
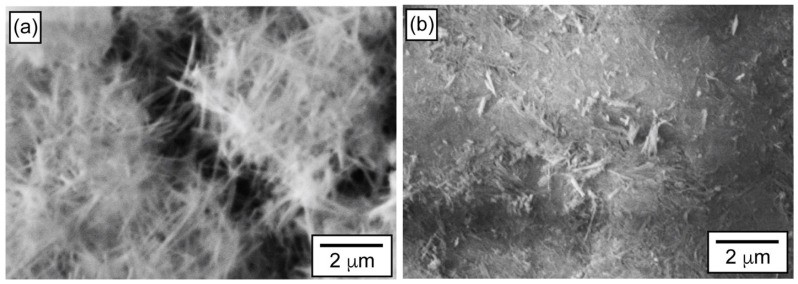
SEM images of the (**a**) HAp powders and (**b**) HAp-P3 pellet.

The XRD patterns of the HAp powders confirm the formation of a hexagonal crystal structure, in agreement with the reference pattern (PDF #09-0432) (Figure 1a). The compacted HAp pellets (Figure 1c–e) retain the characteristic diffraction pattern of hexagonal hydroxyapatite, confirming phase preservation after pressing. However, noticeable changes in relative peak intensities are observed compared to the as-synthesized powder. In particular, the relative intensities of the (002) and (211) diffraction peaks decrease progressively with increasing compaction load, indicating a loss of long-range crystalline order. This behavior is consistent with the development of slight amorphization induced by mechanical compaction. Such amorphization is attributed to lattice disorder generated by the random fracture and rearrangement of HAp nanofibers during pressing, rather than to a controlled reduction in crystallite size. Figure 2 presents SEM images of the HAp powders and the HAp-P3 pellet. The powders exhibit a network of entangled nanofibers with a high aspect ratio, which is typical of powders prepared using the hydrothermal method [11]. After applying a uniaxial compressive load to form the HAp-P3 pellet, this fibrous arrangement was significantly disrupted. The nanofibers fractured and compacted, resulting in smaller nanocrystals, which clarifies the loss of crystallinity observed in XRD.

### 3.2. Defect-Related Luminescence

**Figure 3:**
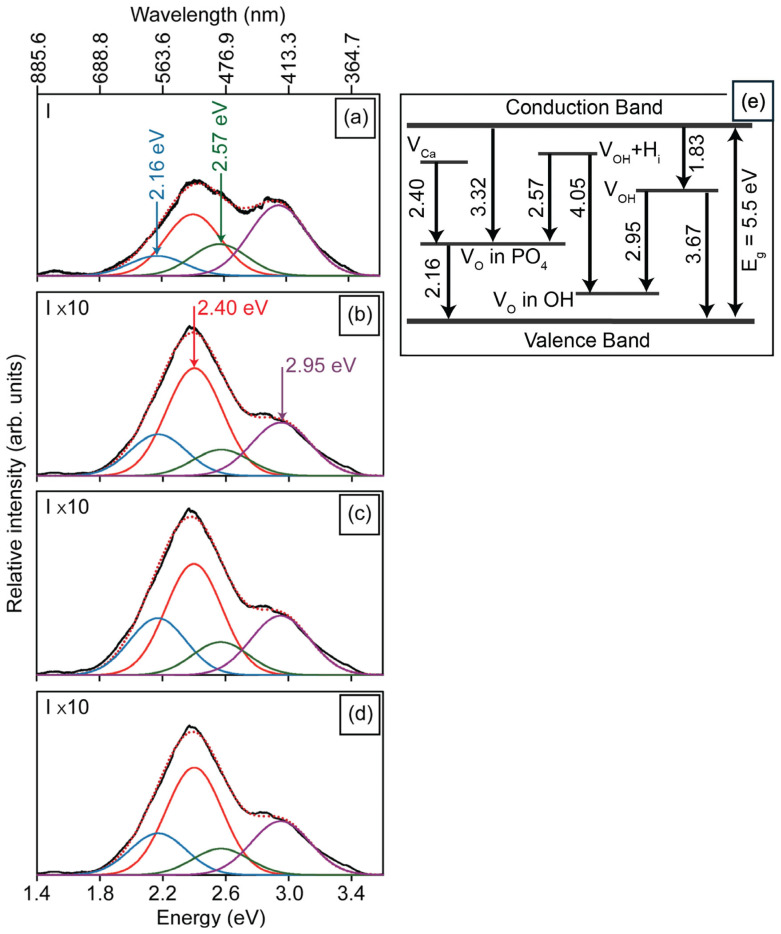
Photoluminescence spectra of the (**a**) HAp powder, and the (**b**) HAp-P3, (**c**) HAp-P4, and (**d**) HAp-P5 pellets, showing the influence of compaction pressure on luminescent properties. The black line represents the experimental spectra. The colored curves correspond to the individual Gaussian components used to deconvolute the emission bands, associated with different defect-related radiative recombination processes, while the red dotted line represents the total fitted spectrum obtained as the sum of all Gaussian contributions. The relative intensity of the pellets is one order of magnitude higher than the intensity of the powders. (**e**) Energy level diagram showing the luminescent emissions of HAp.

To evaluate changes in the defect structure induced by mechanical compression, PL and CL measurements were performed, and are illustrated in Figure 3a–d. The PL spectrum of the HAp powder (Figure 3a) exhibits four main emission bands centered at 2.16, 2.40, 2.57, and 2.95 eV, which are associated with electronic transitions involving oxygen, calcium, and hydroxyl vacancies in the HAp lattice, as illustrated in the energy-level diagram of Figure 3e. These electronic levels were previously reported by our group in hydroxyapatite nanostructures synthesized by the same method [11], where CL, PL, EPR, and DFT data were used to establish the relative positions of intrinsic vacancy levels within the ~5.5 eV band gap. Since both the powders and compacted pellets correspond to Ca-deficient hexagonal HAp, no significant difference in band gap is expected between them. Specifically, the 2.16 eV band arises from transitions between V_O_ levels in the $PO_{4}^{3-}$ groups and the valence band, while the 2.40 eV emission is attributed to transitions between V_Ca_ and V_O_ in the $PO_{4}^{3-}$ groups. The 2.57 eV band originates from transitions between the V_OH_ + H_i_ complex and V_O_ in the $PO_{4}^{3-}$ groups, whereas the 2.95 eV emission is linked to transitions between V_OH_ and V_O_ in ${\mathrm{OH}}^{-}$ groups [24]. Figure 3b–d illustrate the PL intensity of the 2.57 and 2.95 eV emissions, decreasing with increasing compression load for the HAp-P3, HAp-P4, and HAp-P5 samples. This reduction is attributed to a lower density of the defects responsible for these emissions, likely resulting from the filling of V_O_ in ${\mathrm{OH}}^{-}$ and $PO_{4}^{3-}$ groups by atmospheric oxygen incorporated during the compaction process. The pressing of the powders during compaction causes heating, as a consequence of the mechanical shear forces (i.e., friction) experienced by the powders, thus promoting temperature-induced oxygen diffusion.

**Figure 4:**
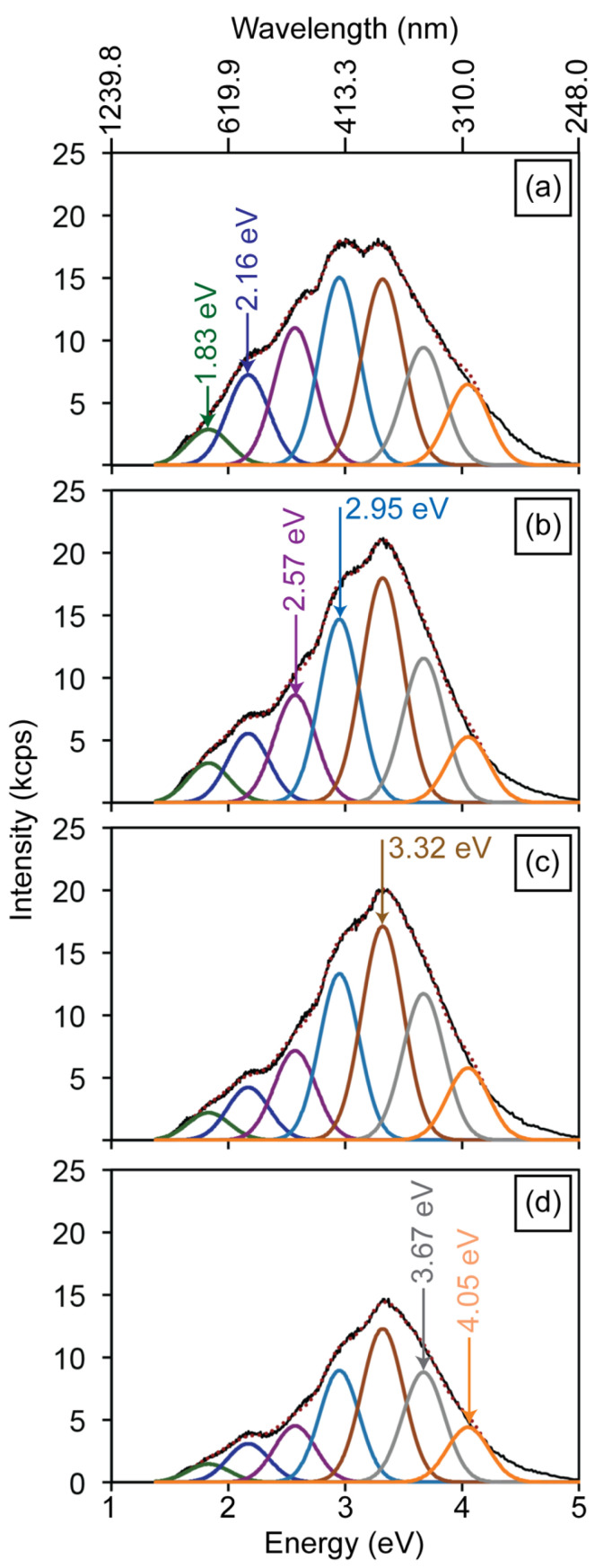
Cathodoluminescence spectra of the (**a**) HAp powder, and the (**b**) HAp-P3, (**c**) HAp-P4, and (**d**) HAp-P5 pellets, highlighting persistent defect-related luminescence. The black line represents the experimental spectra. The colored curves correspond to the individual Gaussian components used to deconvolute the emission bands, associated with different defect-related radiative recombination processes, while the red dotted line represents the total fitted spectrum obtained as the sum of all Gaussian contributions. The maximum values of the deconvolution components for graphs (**a**–**d**) are defined by the energy transitions shown in Figure 3e.

Figure 4a illustrates the CL spectrum obtained from the HAp powders, which is composed of seven emission bands centered at 1.83, 2.16, 2.57, 2.95, 3.32, 3.67, and 4.05 eV. The 1.83 eV emission is attributed to electronic transitions between the conduction band and hydroxyl vacancies (V_OH_). The 3.32 eV emission arises from transitions between the conduction band and defect levels associated with V_O_ in the $PO_{4}^{3-}$ groups, whereas the 4.05 eV band is attributed to transitions involving V_OH_ + H_i_ complexes and V_O_ in ${\mathrm{OH}}^{-}$ groups (Figure 3e). Figure 4b–d present the CL spectra of the HAp-P3, HAp-P4, and HAp-P5 pellets. Consistent with the PL results, these spectra reveal a progressive decrease in the relative intensity of the 2.57 and 2.95 eV bands as the compaction load increases. In addition, a reduction is also observed in the 2.16 eV emission. Considering that these three components are related to the presence of V_O_ in $PO_{4}^{3-}$ and ${\mathrm{OH}}^{-}$ groups within the HAp lattice, their decreasing intensity is attributed to partial filling by atmospheric oxygen incorporated during the mechanical energy applied in the compaction process, consistent with the PL observations.

**Figure 5:**
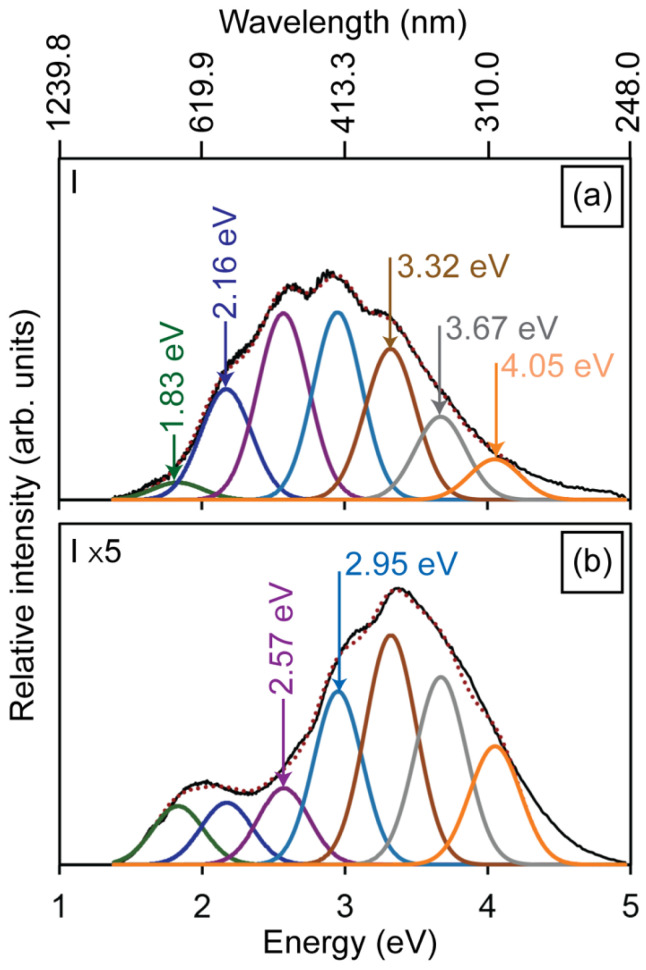
Cathodoluminescence spectra of the (**a**) as-synthesized and (**b**) annealed HAp powder in O_2_ atmosphere at 773 K for 5 h, revealing a decrease in the 2.16 and 2.57 eV bands. The *y*-axis of (**b**) is five times the *y*-axis of (**a**). The black line represents the experimental spectra. The colored curves correspond to the individual Gaussian components used to deconvolute the emission bands, associated with different defect-related radiative recombination processes, while the red dotted line represents the total fitted spectrum obtained as the sum of all Gaussian contributions.

To further validate this interpretation, an additional annealing treatment was performed on as-synthesized HAp powders in flowing O_2_ (50 sccm) at 773 K for 5 h. As shown in Figure 5, the normalized CL spectrum after annealing (Figure 5b) exhibits a drastic reduction in the 2.16 and 2.57 eV emissions, together with a moderate decrease in the 2.95 eV band. These results confirm that oxygen vacancies in the $PO_{4}^{3-}$ groups are particularly unstable, being preferentially filled under an oxidizing environment. Notably, the persistence of the higher-energy bands (3.32, 3.67, and 4.05 eV) after annealing demonstrates their association with more stable oxygen-related complexes, which are less sensitive to oxygen incorporation. The combined evidence from Figure 4 and Figure 5 thus establishes that while mechanical compaction induces partial oxygen incorporation through mechanical-assisted activation, thermal annealing in O_2_ provides a more efficient and selective pathway for the annihilation of the least stable oxygen vacancies in the HAp lattice.

### 3.3. Low-Energy Electron Beam Irradiation

**Figure 6:**
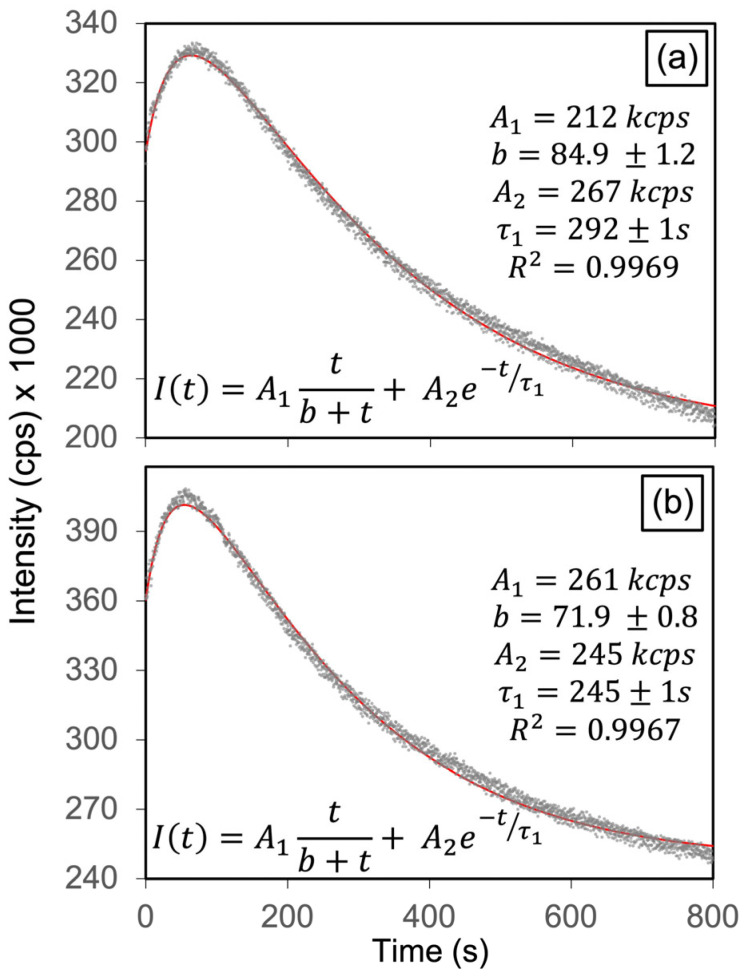
Time-resolved panchromatic CL curves obtained from the HAp-P3 pellet under electron irradiation with current densities of: (**a**) 1.0 × 10^−11^ A/mm^2^, and (**b**) 2.2 × 10^−11^ A/mm^2^. The gray dots represent the experimental data, while the red solid line corresponds to the fitted curve obtained using the analytical model described in the text.

Figure 6 presents the transient panchromatic CL intensity curves of the HAp-P3 pellet under electron beam irradiation at current densities of 1.0 × 10^−11^ A/mm^2^ and 2.2 × 10^−11^ A/mm^2^, respectively. In both cases, the CL intensity initially increases, reaching a maximum at ~60 s for the lower current density (Figure 6a) and ~50 s for the higher one (Figure 6b), followed by a gradual decay. These results indicate that electron irradiation promotes the activation of radiative point defects in HAp, with the rate of defect generation and the saturation time strongly dependent on the applied current density. The irradiation-induced evolution of the CL was modeled using a Monod-type activation and an exponential annealing term:

(1) $$I\left(t\right)=A_{1}\frac{t}{b+t}+A_{2}e^{-t/\tau_{1}}$$

where *A*_1_ and *A*_2_ are amplitude constants, *b* is the half-saturation time in the Monod-type activation term [25], and *t*_1_ is the decay constant in the exponential annealing term. The two-component kinetic model applied here, combining a Monod-type activation with exponential quenching, has precedent in irradiation studies of oxide ceramics [26]. In this framework, the Monod-type term describes the progressive activation or growth of luminescent centers, while the exponential term accounts for their annealing or decay, highlighting the dynamic competition between these two processes captured by the model. The parameter *b* controls the initial activation rate (*A*_1_/*b*). Fits (R^2^ ≈ 0.997) yield *b* = 85 s at 1.0 × 10^−11^ A/mm^2^ and *b* = 24.8 s at 2.2 × 10^−11^ A/mm^2^, indicating a ~4.2-fold faster saturation under higher current density. The corresponding decay constant values, *t*_1_ = 292 s and 245 s, for Figure 6a,b, respectively, indicate slightly faster annealing under stronger irradiation.

**Figure 7:**
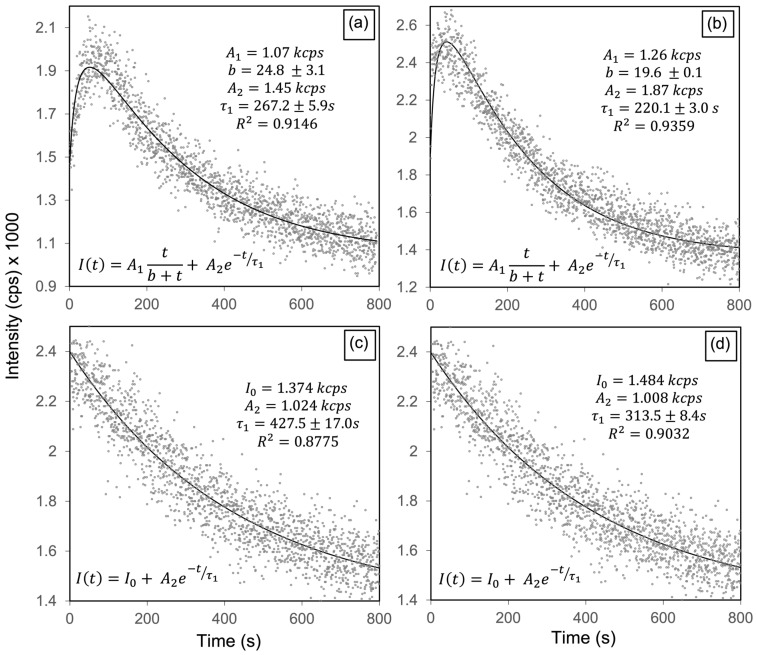
Time-resolved monochromatic cathodoluminescence curves of HAp-P3 pellet for emission energies of (**a**) 2.57, (**b**) 2.95, (**c**) 3.32, and (**d**) 3.67 eV under electron irradiation (*J* = 2.2 × 10^−11^ A/mm^2^). ${\mathrm{OH}}^{-}$ related emissions at 2.57 eV (V_OH_ + H_i_) and 2.95 eV (V_O_ in ${\mathrm{OH}}^{-}$) exhibit rise–decay behavior (electron-stimulated activation followed by annealing). The 3.32 eV band (CB → V_O_ in $PO_{4}^{3-}$) and the 3.67 eV band (V_OH_ → VB, ${\mathrm{OH}}^{-}$ related) show monotonic exponential quenching.

Monochromatic CL decay curves acquired at a current density of 2.2 × 10^−11^ A/mm^2^ for emission components centered at 2.56, 2.95, 3.32, and 3.67 eV, are presented in Figure 7a–d. The 2.57 and 2.95 eV bands exhibit a characteristic rise followed by decay, consistent with the generation and subsequent quenching of radiative centers under electron irradiation. This rise can be interpreted as an activation stage, during which injected electrons accumulate within the HAp lattice, enhancing its internal electric field and polarization [12]. Under these conditions, positively charged OH-channel vacancies (V_OH_ and V_OH_ + H_i_) experience an increased electron-capture cross-section, resulting in a higher population of radiative active charged states responsible for the 2.57 and 2.95 eV emissions. Once these charged states are saturated, dynamic annealing progressively reduces the population of radiative sites, producing the observed decay. In contrast, the 3.32 and 3.67 eV components display only a monotonic exponential decay, indicating that the corresponding centers are not activated, but undergo progressive annealing. This behavior suggests that the 3.67 eV emission (V_OH_ → VB) is hole-limited, since radiative recombination at this transition requires the participation of holes in the valence band. Under LEEBI conditions, the continuous injection of electrons shifts the Fermi level upward, reducing the hole concentration available for recombination, thereby suppressing the emission. The combined effects of charge accumulation and lattice polarization thus explain the selective activation of only certain OH-channel vacancies, which can efficiently capture the injected electrons during the early stage of LEEBI because of their positive charge.

Table 2 presents the decay times and corresponding annealing times for the four emissions, showing that the OH-related bands at 2.57 and 2.95 eV, which display rise–decay behavior under LEEBI, have the shortest τ_1_ values, indicating that these activated OH-channel vacancies anneal more rapidly than the other monitored defect centers. These results demonstrate the selective activation of defect-related emissions in HAp, with only specific OH-channel vacancies (2.57 and 2.95 eV) becoming radiatively active under LEEBI. The panchromatic kinetics (Figure 6) and band-resolved dynamics (Figure 7) confirm a defect-selective response to electron irradiation. Based on the electronic transition diagram in Figure 3e, this selectivity arises from charge-state-dependent activation of OH-channel vacancies, whereas the 3.32 and 3.67 eV bands exhibit only annealing-driven decay (Figure 7c,d). These findings reveal that the activation of OH-channel vacancies results from electron-stimulated enhancement of their radiative efficiency, driven by charge accumulation and polarization effects within the HAp lattice. Notably, selective activation of oxygen-vacancy luminescence under electron injection has been reported in wide-bandgap oxides such as ZnO and TiO_2_ [27,28]. Furthermore, Figure 7 and Table 2 show that OH-channel vacancy emissions at 2.57 and 2.95 eV follow a rise–decay behavior with shorter decay constants (t_1_ ≈ 220–270 s), corresponding to higher electron-stimulated annealing rates (1/*t*_1_), than the bands at 3.32 (CB → V_O_ in $PO_{4}^{3-}$) and 3.67 eV (V_OH_ → VB). Thus, while LEEBI efficiently activates OH-channel vacancies, these radiative states also anneal more rapidly than the 3.32 and 3.67 eV emissions, where the latter show a single-exponential decay with no activation step. Under oxidizing conditions (Figure 5), vacancies are preferentially eliminated by oxygen incorporation, producing a marked decrease in the 2.16 and 2.57 eV bands and a moderate reduction at 2.95 eV. Taken together, these high 1/t_1_ values for the 2.57 and 2.95 eV bands indicate faster electron-stimulated annealing of OH-channel vacancies, whereas O_2_ annealing more efficiently suppresses V_O_ in ${\mathrm{OH}}^{-}$ and $PO_{4}^{3-}$ groups.

### 3.4. Gallium Ion Irradiations

**Figure 8:**
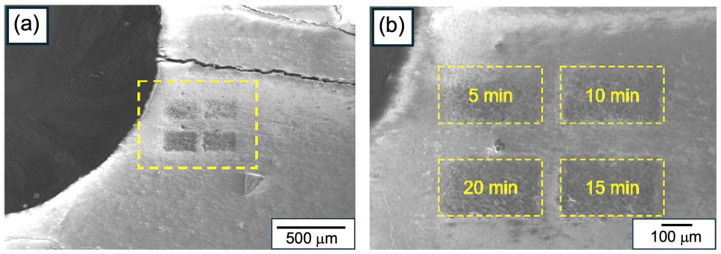
(**a**) Panoramic scanning electron micrographs of HAp-P3 pellet surface and (**b**) detail of four regions irradiated with ${\mathrm{Ga}}^{+}$ ions (*J* = 1.4 × 10^−13^ A/mm^2^) for 5, 10, 15, and 20 min.

**Figure 9:**
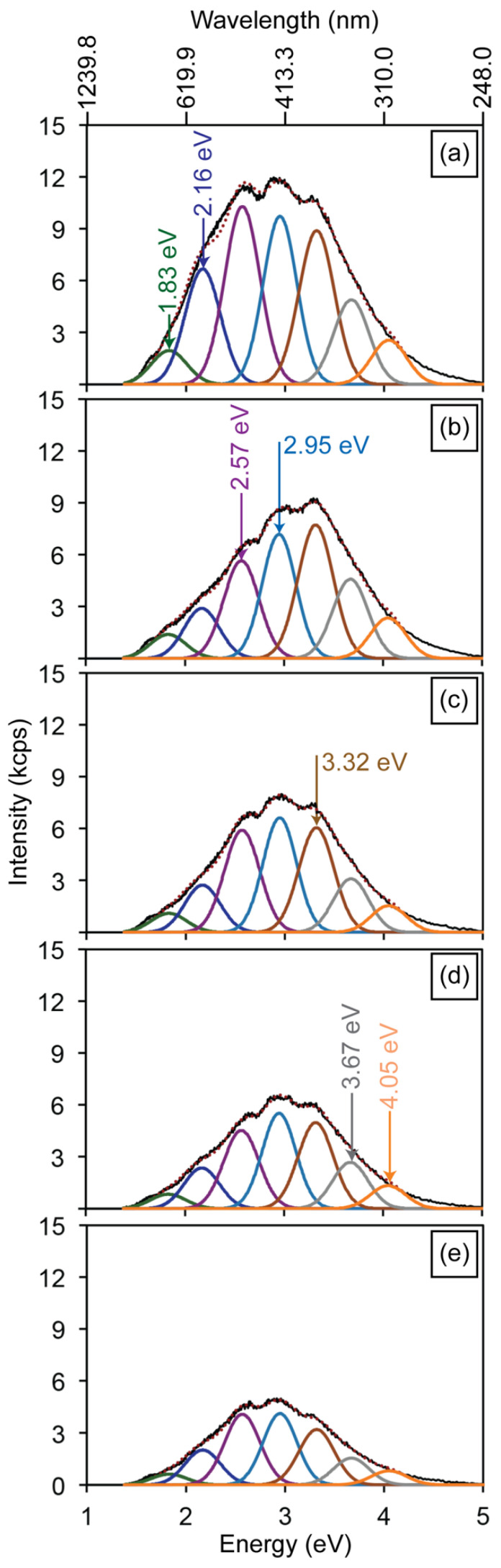
Cathodoluminescence spectra of the HAp-P3 pellet acquired after ${\mathrm{Ga}}^{+}$ irradiation times of (**a**) 0 min, (**b**) 5 min, (**c**) 10 min, (**d**) 15 min, and (**e**) 20 min, showing the progressive quenching of defect-related luminescence. The black line represents the experimental spectra. The colored curves correspond to the individual Gaussian components used to deconvolute the emission bands, associated with different defect-related radiative recombination processes, while the red dotted line represents the total fitted spectrum obtained as the sum of all Gaussian contributions.

To complement the electron-irradiation results, and to evaluate the annealing effect of ${\mathrm{Ga}}^{+}$ ion irradiation on the CL emission in HAp, the HAp-P3 sample was exposed to increasing irradiation doses. Figure 8a,b show SEM images of the irradiated regions, a panoramic view of the pellet surface (Figure 8a) and a magnified detail of four selected areas (Figure 8b). These regions were irradiated at a current density of 1.4 × 10^−13^ A/µm^2^ for exposure times of 5, 10, 15, and 20 min. The corresponding CL spectra, presented in Figure 9a–e, reveal a progressive quenching of the HAp luminescence, consistent with an annealing effect [29].

Figure 9a shows the reference CL spectrum prior to ${\mathrm{Ga}}^{+}$ exposure, whereas Figure 9b illustrates the response after 5 min of irradiation. A general reduction in CL intensity is observed across all components, with the most pronounced quenching occurring in the 2.15 and 2.57 eV bands. As previously discussed, both of these bands originate from electronic transitions involving V_O_ in $PO_{4}^{3-}$ groups (see Figure 3e), suggesting that this type of defect is structurally less stable than other point defects in the HAp lattice. At longer exposure times (Figure 9c–e), all emission components exhibit a monotonic decrease in intensity, reflecting a more homogeneous annealing process throughout the probed volume ${\mathrm{Ga}}^{+}$ ion beams. Under irradiation, dynamic annealing takes place as defects recombine, reducing their density and quenching the associated luminescence centers [30,31].

**Figure 10:**
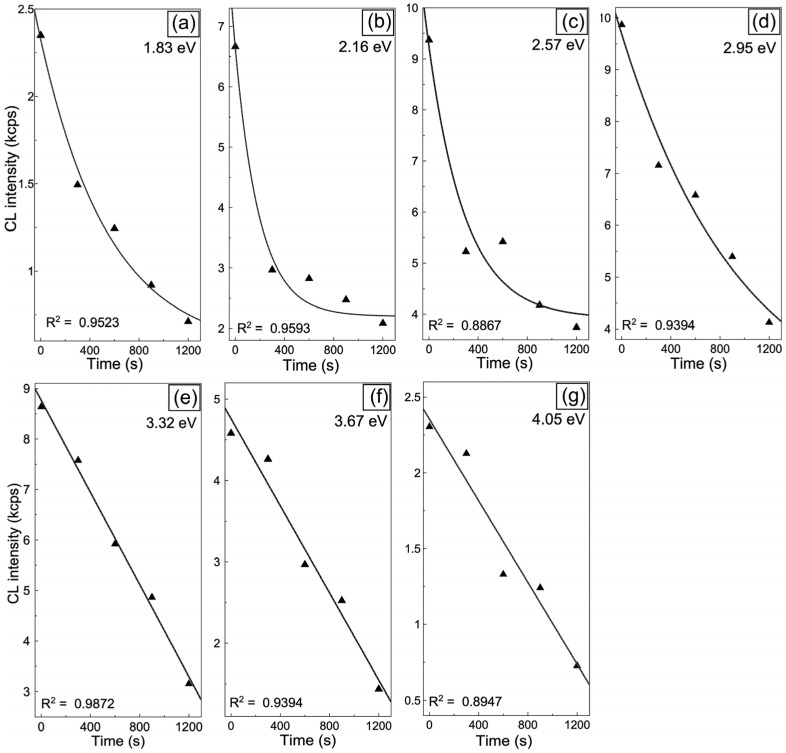
Cathodoluminescence decay curves of emissions at (**a**) 1.83, (**b**) 2.16, (**c**) 2.57, (**d**) 2.95, (**e**) 3.32, (**f**) 3.67, and (**g**) 4.05 eV after ${\mathrm{Ga}}^{+}$ irradiation of HAp-P3 pellet, indicating defect-specific stability and quenching behavior. The triangular symbols represent the experimental decay data, while the solid lines correspond to the fitted curves obtained using the decay model described in the text.

The evolution of the maximum CL intensity with irradiation time is summarized in Figure 10. The decay of the 1.83, 2.16, 2.57, and 2.95 eV bands follows an exponential trend, I = A exp(−tτ), with characteristic decay times τ of approximately 560, 198, 300, and 980 s, respectively. In contrast, the 3.32, 3.67, and 4.05 eV exhibit an approximately linear decay, indicating a steady decrease in emission intensity over time. These results demonstrate that the centers responsible for the 2.16 and 2.57 eV emissions are annealed more rapidly (higher 1/τ values) than other defect-related centers, consistent with their lower lattice stability. This interpretation is further supported by the O_2_ annealing experiment (Figure 5), which shows a marked suppression of the same two emissions. Since both bands are associated with electronic transitions involving V_O_ in $PO_{4}^{3-}$ groups, these findings demonstrate that such vacancies are the most unstable defects in the HAp lattice, readily annihilated through oxygen incorporation during ${\mathrm{Ga}}^{+}$ ion irradiation. The decay time values can be quantitatively expressed as τ_2.16_ ≈ 198 s < τ_2.57_ ≈ 300 s < τ_1.83_ ≈ 560 s < τ_2.95_ ≈ 980 s, and thus follow an increasing trend, confirming that oxygen vacancies in $PO_{4}^{3-}$ groups are the least stable, whereas OH-channel vacancies exhibit the highest stability under ${\mathrm{Ga}}^{+}$ irradiation. Overall, ${\mathrm{Ga}}^{+}$ irradiation not only quenches luminescence but also selectively anneals the most unstable oxygen-related defects, V_O_ in $PO_{4}^{3-}$ groups. In contrast, electron-beam irradiation (Figure 7 and Figure 8) promotes the activation of ${\mathrm{OH}}^{-}$ related vacancy complexes. In summary, these findings highlight two distinct pathways for defect evolution in HAp that depend on the irradiation source, either luminescence activation under electron irradiation or suppression under ion irradiation.

## 4. Conclusions

This study demonstrates that hydroxyapatite (HAp) exhibits distinct, defect-selective luminescence behaviors under electron and ion irradiation, thereby revealing differences in vacancy stability within the HAp lattice. Low-energy electron beam irradiation (LEEBI, 15 keV) selectively enhances the radiative efficiency of OH-channel vacancies (V_OH_ and V_OH_ + H_i_), producing transient luminescence that can be quantitatively described by competing activation and annealing kinetics. This activation arises from electron-stimulated charge accumulation and polarization effects that increase the electron-capture cross-section of positively charged vacancy states. In contrast, ${\mathrm{Ga}}^{+}$ ion irradiation (30 keV) produces progressive luminescence quenching, consistent with selective annealing of the least stable oxygen-related vacancies in $PO_{4}^{3-}$ groups. Together, these complementary behaviors establish irradiation as a versatile tool for defect engineering in HAp, enabling either the selective activation or suppression of specific vacancy species. The ${\mathrm{OH}}^{-}$ channel vacancy activation under LEEBI and the defect-quenching pathway under ${\mathrm{Ga}}^{+}$ ions reveal the relative stability of intrinsic vacancies in HAp. These complementary irradiation routes provide a defect-selective framework for engineering the optical response of hydroxyapatite, enabling targeted activation or suppression of specific vacancy populations. Beyond their fundamental significance, these results open opportunities for tailoring the optical, sensing, and bioimaging functionalities of hydroxyapatite nanostructures through controlled irradiation strategies.

## Figures and Tables

**Table 1:** Elemental composition measured by EDS and XPS of the HAp powder sample. The Ca/P atomic ratio confirms the Ca-deficient stoichiometry of the synthesized hydroxyapatite.

**Powder Sample**	**EDS (at.%)**			**EDS Ca/P** **Atomic Ratio**
	O	Ca	P	
**HAp**	71.04	16.23	12.74	1.27
	**XPS (at.%)**			**XPS Ca/P** **Atomic Ratio**
	O 1s	Ca 2p	P 2p	
	56.31	24.48	19.21	1.27

**Table 2:** Decay constants t_1_ and corresponding annealing rates 1/τ_1_ extracted from the fits in Figure 7 (*J* = 2.2 × 10^−11^ A/mm^2^). Under electron irradiation, the OH-related bands at 2.57 and 2.95 eV display shorter t_1_ (higher 1/τ_1_), compared to the 3.32 eV $PO_{4}^{3-}$-related band (CB → V_O_ in $PO_{4}^{3-}$), and the OH^−^ related band at 3.67 eV (V_OH_ → VB).

Emission Energy (eV)	Electronic Transition	τ_1_ (s)	1/τ_1_ (10^−3^ s^−1^)
2.57	V_OH_ + H_i_ → V_O_ in PO_4_	267.0 ± 6.0	3.75 ± 0.08
2.95	V_OH_ → V_O_ in OH	220.1 ± 3.0	4.54 ± 0.06
3.32	CB → V_O_ in PO_4_	427.5 ± 17.0	2.34 ± 0.09
3.67	V_OH_ → VB	313.5 ± 8.4	3.19 ± 0.09

## Data Availability

The original contributions presented in this study are included in the article. Further inquiries can be directed to the corresponding authors.
